# Dinner to Dr. Roosa

**Published:** 1904-03

**Authors:** 


### Dinner to Dr. Roosa.

DR. D. B. St. John Roosa, of New York, was the guest of honor at a dinner tendered to him by distinguished citizens, physicians, and laymen residing in various parts of the country, on Tuesday evening, March 1, 1904. Special importance is given to the function because it also commemorated the twenty-one years since the inauguration of post-graduate medical instruction in this country, Dr. Roosa being the founder of the first school of that kind on this side of the Atlantic. The dinner, which was served at Delmonico's, was presided over by Dr. William Osler, of Baltimore, and was attended by a large number of representative physicians and business men at home and from a distance, all the leading cities being represented. It was an event worthy the occasion in every way and served to accentuate the respect and affection of the people all over the country for the distinguished guest of honor. The following names were appended to the cards of invitation: Drs. William T. Bull, Francis Delafield, Bache Emmet, Virgil P. Gibney, Edward G. Janeway, William M. Polk, A. A. Smith, A. H. Smith, and Robert F. Weir, New York; Albert Vander Veer, Albany; A. W. Calhoun, Atlanta; William H. Welch, William S. Halsted, Baltimore; Clarence J. Blake, Maurice H. Richardson, Boston; William W. Keen, John H. Musser, J. C. Wilson, Philadelphia; George Ben Johnston, Richmond; William J. Mayo, Rochester, Minn.; and James W. Alexander, Edward T. Bartlett, Cornelius N. Bliss, William D. Howells, Morris K. Jessup, Edward King, John E. Parsons, J. Edward Simmons, and J. H. Van Amringe, of New York.

The guest of honor, noted as a gifted speaker, made one of the best speeches of his life, and other addresses in timely keeping with the event, served to make it one of the most enjoyable of occasions as well as one of the most notable dinners that has ever been tendered a physician in this country.

